# Segmental ureteroileal conduit resection for the treatment of distal upper urinary tract recurrence of bladder cancer following cystectomy

**DOI:** 10.1186/s40880-015-0077-8

**Published:** 2016-01-21

**Authors:** Shu-Xiong Zeng, Xin Lu, Wei-Dong Xu, Zhen-Sheng Zhang, Hai-Hang Li, Ying-Hao Sun, Chuan-Liang Xu

**Affiliations:** Department of Urology, Changhai Hospital, Second Military Medical University, Shanghai, P. R. China; Department of Burns, Changhai Hospital, Second Military Medical University, Shanghai, P. R. China

**Keywords:** Cystectomy, Urothelial carcinoma, Upper urinary tract recurrence, Segmental resection

## Abstract

Segmental ureterectomy is less invasive than radical nephroureterectomy and results in nephron preservation and satisfactory tumor control. This study was to determine the feasibility of segmental ureteroileal conduit resection (SUICR) for patients with distal upper urinary tract recurrence of bladder cancer following radical cystectomy. Four patients with high-grade distal upper urinary tract recurrence underwent SUICR 15–108 months after radical cystectomy. The surgical technique details of SUICR, operative results, and follow-up outcomes are reported. The median operation time was 280 min, and estimated blood loss was less than 100 mL. One patient suffered from ileus 5 days after surgery and was managed conservatively. Histopathologic evaluation showed high-grade stages pTa-pT1 diseases for these patients, and ureteral margins were all negative. No patient suffered from tumor recurrence, with a median follow-up of 39 months. SUICR preserved the ipsilateral renal unit and conformed to oncological principles during surgery. The oncological outcome was satisfactory for these properly selected patients. This technique provides a valid alternative to nephroureterectomy for patients with imperative indications and high-grade upper urinary tract recurrence of bladder cancer following radical cystectomy.

## Background

Upper urinary tract recurrence of bladder cancer following radical cystectomy is extremely rare, with a reported incidence ranging from 0.75% to 6.4% after cystectomy [1, 2]. The characteristics of the clinical course of upper urinary tract recurrence are thus not yet fully understood. As urothelial carcinoma is a multifocal disease affecting the entire urothelium, radical nephroureterectomy (RNU) with bladder cuff excision is the standard treatment for urothelial carcinoma of the upper urinary tract regardless of the location of the tumor because it eliminates the risk of ipsilateral recurrence [1]. However, RNU increases the risk of renal function decline and cardiovascular events and affects the eligibility for adjuvant chemotherapy. Furthermore, patients undergoing RNU are still at life-long risk of upper urinary tract recurrence in the remaining renal unit [2, 3]. As a result, renal-sparing surgeries for patients with urothelial carcinoma of the upper urinary tract, e.g., segmental ureterectomy, laser endoscopic ablation, and percutaneous tumor ablation, have been developed for patients under certain conditions [4–7]. The long-term survival rates of patients after RNU and renal-sparing surgery were comparable in several retrospective studies with long-term follow-up [3, 5, 8, 9]. In addition, renal-sparing management is more cost-effective than RNU [10]. Even for patients with a normal contralateral kidney, by sparing the ipsilateral kidney, more patients will be eligible for and benefit from adjuvant or salvage chemotherapy if it is required [6, 11].

Segmental ureterectomy is recommended by The National Comprehensive Cancer Network guidelines for properly selected patients, e.g., renal insufficiency, solitary functional kidney, and low-grade middle or distal ureteral tumors [12]. Unlike other conservative treatments of upper urinary tract recurrence, such as endoscopic ablation, segmental ureterectomy is the only renal-sparing procedure that enables intraoperative analysis of excision margins and accurate histopathologic examination, both of which are crucial for predicting prognosis [6, 8]. Furthermore, the largest retrospective series involving 2044 patients showed that the 5-year cancer-specific survival rate was comparable between segmental ureterectomy and RNU groups, suggesting that segmental ureterectomy did not undermine cancer control outcomes [7]. This result was also confirmed by several other multicenter retrospective studies [3, 8, 11].

To the best of our knowledge, there are few studies applying segmental ureterectomy for patients with upper urinary tract recurrence of bladder cancer following radical cystectomy, as this is an extremely rare disease requiring proficient surgical technique. The present study aimed to introduce the technique of segmental ureteroileal conduit resection (SUICR) for the treatment of distal upper urinary tract recurrence of bladder cancer following radical cystectomy.

## Case report

Between June 2009 and March 2014, four patients were diagnosed with high-grade upper urinary tract recurrence in the distal ureter (defined as the distal 1/3 of the ureter) following radical cystectomy for bladder cancer and underwent SUICR. Individual characteristics of the patients and features of upper urinary tract recurrence are summarized in Table 1. No patient received chemotherapy or radiotherapy previously. The preoperative evaluation included intravenous urography, computed tomography, magnetic resonance urography, flexible cystoscope, urinary cytology, and serum creatinine detection. Ureteroscopy was performed for tumor biopsy, and several steps were taken to avoid tumor implantation in the upper ureter, i.e., a low water injection pressure, low-dose diuretics used during operation, and protecting the healthy urothelium from injury. Indications for SUICR included solitary kidney or impaired renal function caused by hydroureteronephrosis. Operations were performed after detailed information about SUICR was provided to patients and informed consent was obtained. Because all interventions given were part of normal health care, ethical approval was not required.

**Table 1:** Clinical features of four patients with distal upper urinary tract recurrence of bladder cancer following cystectomy

Patient	Age (years)	Gender	Pathologic result of radical cystectomy^a^	Duration from cystectomy to recurrence (months)	Recurrence location^b^	Reasons for tumor detection	Indications for SUICR	Serum creatinine (μmol/L)	
								Preoperative	Postoperative^c^
1	73	Male	G3 with CIS, T1N0M0	34	Right, distal	Microscopic hematuria	Solitary kidney	132	129
2	68	Male	G3, T2N0M0	28	Right, distal	Hematuria	Solitary kidney	110	116
3	64	Male	G3, T1N0M0	15	Left, distal	Hematuria and positive urinary cytology	Solitary kidney	96	101
4	66	Male	G3, T2N0M0	108	Right, distal	Hematuria and positive urinary cytology	Declined renal function caused by hydronephrosis	354	136

*SUICR* segmental ureteroileal conduit resection; *CIS* carcinoma in situ

^a^Tumor staging and grading were based on the 2009 International Union Against Cancer TNM classification and the 2004 World Health Organization system. G3 = high-grade

^b^Distal ureter was defined as the distal 1/3 of the ureter

^c^Postoperative creatinine was examined 5–7 days after surgery

**Fig. 1:**
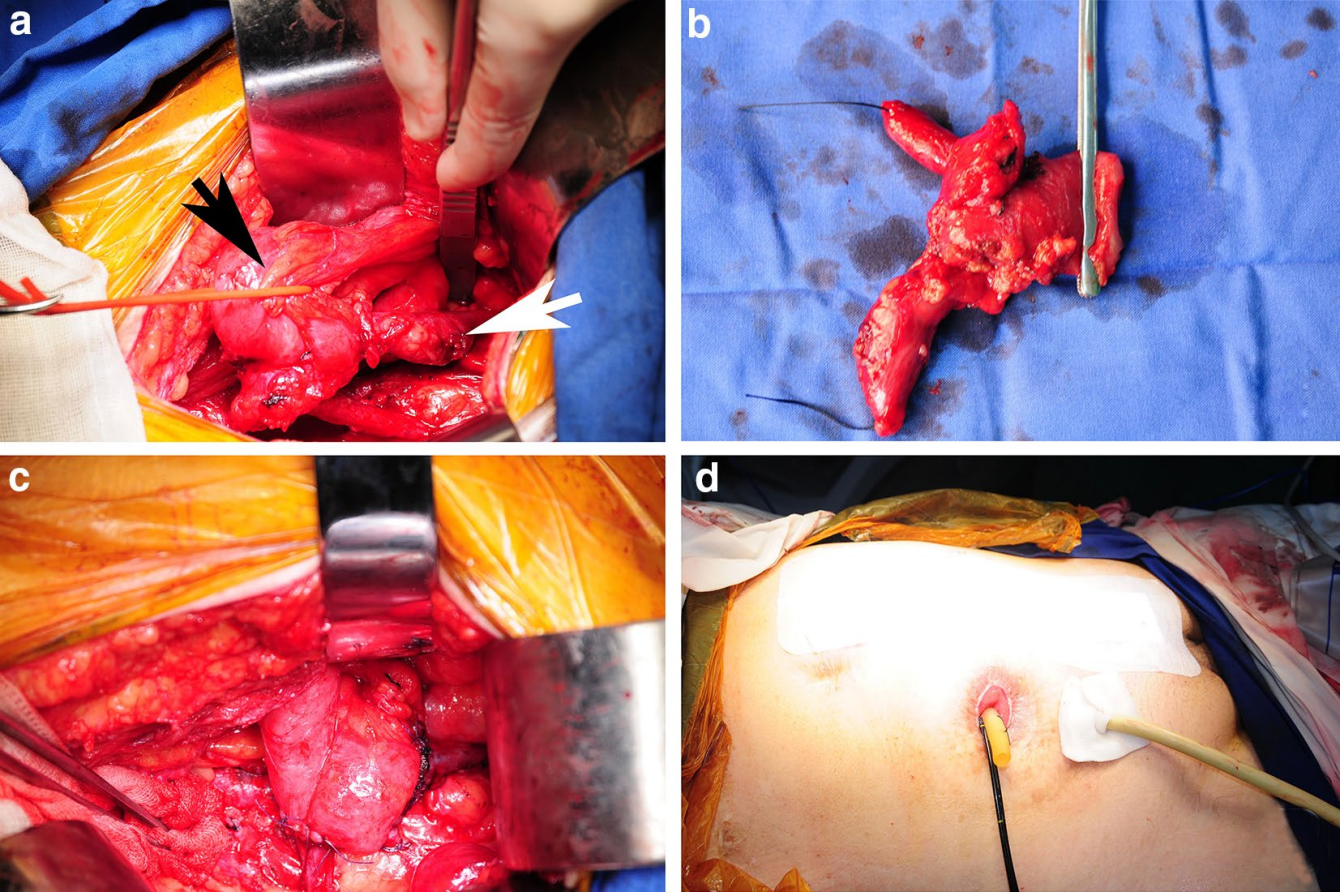
Segmental ureteroileal conduit resection procedures. **a**. The ileal conduit (*black arrow*) and the affected ureter (*white arrow*) were exposed. **b**. The ureters and ileal conduit were segmentally removed. **c**. The transected ureter was reanastomosesed to the residual ileal conduit. **d**. Drainage catheters were placed in the lower abdomen and stoma

**Fig. 2:**
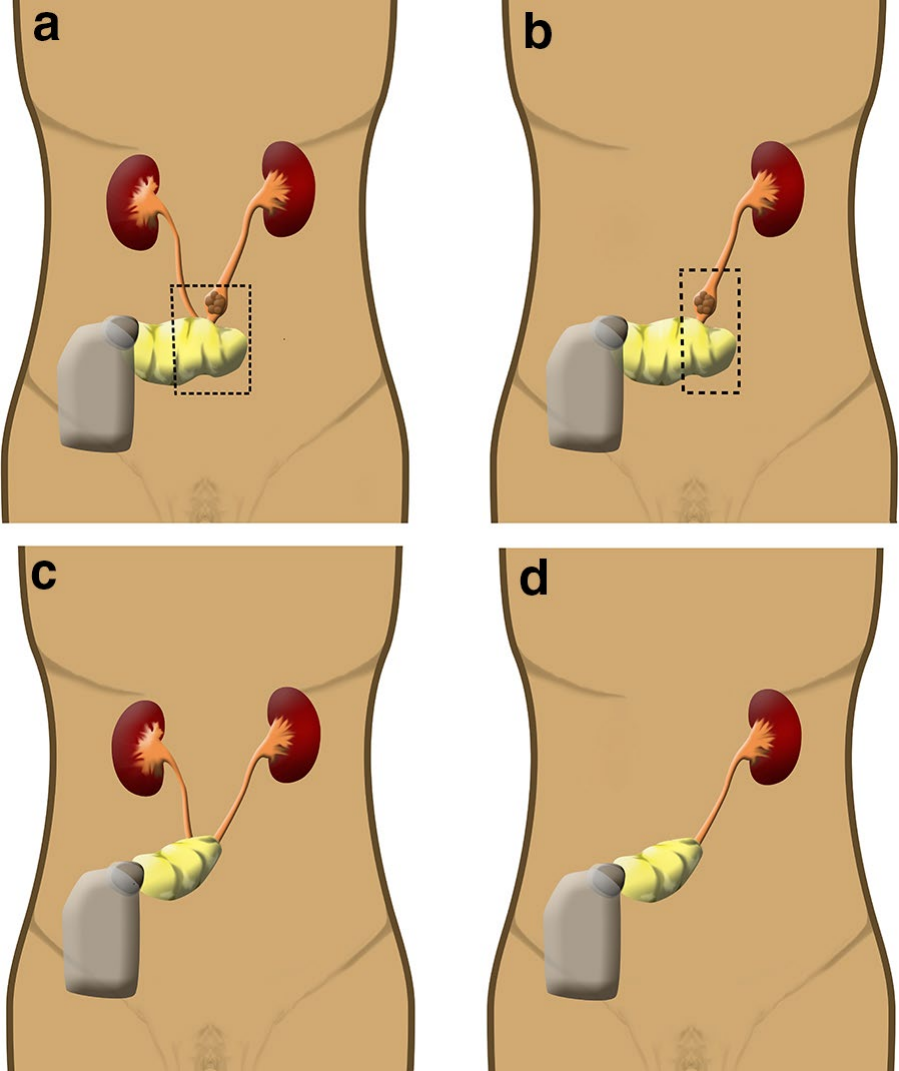
Schematic drawing of the segmental ureteroileal conduit resection technique. **a** and **b**, The tumor to be segmentally removed (*indicates by the dashed line*). **c** and **d**, Patients after segmental ureteroileal conduit resection operation

Under general anesthesia, the patients were placed in a supine position. An approximate 15-cm low-abdominal incision was made in the previous cystectomy incision site. Due to previous open surgery, extensive enterolysis with sharp dissection was performed first, and care needed to be taken not to compromise the intestine. Surgeons would then have good access to the ileal conduit and ureter (Fig. 1a). The lysis of the ileal conduit from local adhesions was then performed, and the ureter was carefully dissected from beyond the bifurcation of the iliac vessels to the urinary diversion. The affected ureter was ligated 2 cm proximal to the ureter tumor, and direct contact with the tumor was avoided through these procedures. For one patient with hydroureteronephrosis, distal upper urinary tract recurrence was suspected on both sides, and the contralateral ureter was also ligated (Fig. 1b). The ileal conduit was clipped with an intestinal clamp approximately 3–4 cm distal to the ureteroileal conduit anastomosis, and segmental resection of the compromised ureteroileal conduit was then performed (Fig. 1b). Frozen sections of the proximal ureteral margin were generated, and the proximal ureter was resected until the frozen sections were negative. The transected ureter was finally reanastomosed to the ileal conduit by routine procedures in radical cystectomy with ileal conduit diversion as described previously [13]. A single-J ureteral stent was placed before closing the anastomosis (Fig. 1c). Lymphadenectomy was not performed for these patients. Finally, drainage catheters were placed in the lower abdomen and stoma (Fig. 1d). A schematic drawing of SUICR is shown in Fig. 2. Pathologic evaluation of tumor stage and grade were based on the 2009 International Union Against Cancer TNM classification and the 2004 World Health Organization system [14, 15].

SUICR was performed successfully in all four patients without major intraoperative complications. Detailed information on the intra- or postoperative characteristics of the surgery is shown in Table 2. The median operation time was 280 min, and the estimated blood loss was less than 100 mL. The changes in serum creatinine before and after surgery are shown in Table 1. The median time to liquid intake was 3 days, and median hospital stay after surgery was 5.5 days. One patient suffered from ileus 5 days after surgery and was managed conservatively. Frozen sections and histopathologic evaluation of the resected ureter margins were all negative. The histopathologic analysis revealed non-muscle invasive, high-grade urothelial carcinoma in these patients (pTa-pT1). The single-J stent was removed 8 weeks after surgery. No patient received chemotherapy or radiotherapy after SUICR.

**Table 2:** Results of patients who underwent SUICR

Patient	Operation duration (min)	Estimated blood loss (mL)	Complications in hospital	Length of hospital stay (days)	Pathologic result	Survival after SUICR (months)	Long-term complication
1	300	<100	None	6	G3, T1N0M0	66	None
2	240	<100	None	5	G3, TaN0M0	46	Ureteroileal conduit anastomosis stricture
3	320	<100	Ileus	8	G3, T1N0M0	32	None
4	260	<100	None	5	G3, TaN0M0	13	None

Abbreviation as in Table 1. All patients are recurrence-free at present

Postoperative surveillance consisted of urinalysis, serum creatinine detection, and urinary system ultrasonography every 3 months in the first year and every 6 months thereafter. Urinary cytology, abdominal computed tomography, intravenous urography, or magnetic resonance urography was performed every 6 months in the first 3 years and annually thereafter. Flexible cystoscopy and ureteroscopy were only indicated in cases of suspicious urinary cytology and radiological imaging.

Patients were followed for a median of 39 months (range, 13–66 months), and all of them were still alive. No upper urothelial carcinoma recurrence or declined renal function was diagnosed. One patient suffered from ureteroileal conduit anastomotic stricture 11 months after SUICR, and was successfully treated with ureteral stent dilations and retrograde placement of single-J stent guided by flexible cystoscope [16].

## Discussion

In the present study, we conformed to the oncological principles of segmental ureterectomy and modified this technique to treat properly selected patients with upper urinary tract recurrence of bladder cancer following radical cystectomy. The major advantage of this modified technique was to preserve the function of the kidney. The conduit was not completely removed to perform ureterocutaneostomy because the ureter was not long enough to reach tension-free anastomosis after the distal portion was removed. Four patients underwent SUICR. Three of them had a solitary kidney, and one had renal function insufficiency due to hydroureteronephrosis caused by suspicious distal ureter tumor recurrence on both sides. None of these patients developed tumor recurrence, with a median follow-up of 39 months. These results suggest that SUICR is promising for properly selected patients with upper urinary tract recurrence, and it can achieve valid tumor control.

The reported incidence of urothelial tumors of the distal ureter (70%) is higher than that of the middle (25%) or proximal ureter (5%) [17, 18]. Urothelial tumors of the distal ureter were more likely to be solitary, small, and low-risk than those in the upper parts. They recur less frequently, and endoscopic treatment might thus be proposed for low-grade tumors [18]. For high-grade upper urinary tract recurrence, a more aggressive treatment modality is needed. As a result, SUICR might be a valuable alternative to RNU for patients with high-grade distal upper urinary tract recurrence and imperative indications. Selecting patients who would benefit more from this technique remains a challenge, and the technique is generally only recommended for patients with imperative indications. However, Jeldres et al. [7] suggested that segmental ureterectomy might be suitable for all patients with urothelial tumors when it was technically feasible, even including carefully selected patients with stage T3 or T4 disease. In contrast, Li et al. [19] recommended segmental ureterectomy only for patients with unilateral low-grade and noninvasive tumors, as patients who underwent distal ureterectomy had shorter recurrence-free survival compared with patients who underwent RNU.

SUICR is a complex technique because of the extensive adhesion and altered anatomy in the abdomen. To assure a satisfactory outcome, attention needs to be paid to several important points. First, to minimize the risk of tumor spillage, direct contact with the tumor should be avoided, and blockage proximal and distal to the tumor, with at least 2-cm safe margin, should be performed when the ureter and ileal conduit are adequately freed. Second, iliac vessels and intestines should be carefully protected when performing enterolysis and releasing the ureter and ileal conduit from adjacent tissues. Third, to achieve tension-free ureteroileal conduit anastomosis, the ureter should be freed as long as possible, and a new ileal conduit should be constructed if required. Last but not least, as urothelial carcinoma is a multifocal disease, patients should be willing to follow stringent surveillance. Although patients who underwent segmental ureterectomy were advised to adhere to more-aggressive life-long surveillance, the optimum regimen for upper urinary tract recurrence following radical cystectomy remains controversial [20, 21]. The majority of upper urinary tract recurrence was detected based on symptoms rather than radiological or cytological surveillance. Approximately 800 radiological examinations were performed to identify only one patient with upper urinary tract recurrence [21–23]. Limitations of the SUICR technique should be addressed. Although the oncological outcome in our modified technique was satisfactory, only four patients underwent our modified technique, with no control group. This was due to the paucity of upper urinary tract recurrence following radical cystectomy, and RNU was still regarded as the standard treatment. Meanwhile, whether SUICR could be successfully performed also depends on the previous operation style and intraoperative exploration. However, this study primarily introduced an alternative technique for urologists to address some challenging tasks of upper urinary tract recurrence following radical cystectomy, and patients were also likely to benefit from this treatment.

## Conclusions

SUICR preserves the ipsilateral renal unit and conforms to oncological principles during surgery. Although this technique is technically demanding, it is less complex and invasive than RNU. Moreover, the oncological outcome of this technique is satisfactory for properly selected patients. In aggregate, SUICR provides a valid alternative to RNU for patients with imperative indications and high-grade upper urinary tract recurrence of bladder cancer following radical cystectomy.

## References

[1] Rouprêt M, Babjuk M, Comperat E, Zigeuner R, Sylvester RJ, Burger M (2015). European association of urology guidelines on upper urinary tract urothelial cell carcinoma: 2015 update. Eur Urol.

[2] Dalpiaz O, Ehrlich G, Quehenberger F, Pummer K, Zigeuner R (2014). Distal ureterectomy is a safe surgical option in patients with urothelial carcinoma of the distal ureter. Urol Oncol.

[3] Simhan J, Smaldone MC, Egleston BL, Canter D, Sterious SN, Corcoran AT (2014). Nephron-sparing management vs radical nephroureterectomy for low- or moderate-grade, low-stage upper tract urothelial carcinoma. BJU Int.

[4] Bin X, Roy OP, Ghiraldi E, Manglik N, Liang T, Vira M (2012). Impact of tumour location and surgical approach on recurrence-free and cancer-specific survival analysis in patients with ureteric tumours. BJU Int.

[5] Gadzinski AJ, Roberts WW, Faerber GJ, Wolf JS (2010). Long-term outcomes of nephroureterectomy versus endoscopic management for upper tract urothelial carcinoma. J Urol.

[6] Seisen T, Colin P, Roupret M (2015). Risk-adapted strategy for the kidney-sparing management of upper tract tumours. Nat Rev Urol.

[7] Jeldres C, Lughezzani G, Sun M, Isbarn H, Shariat SF, Budaus L (2010). Segmental ureterectomy can safely be performed in patients with transitional cell carcinoma of the ureter. J Urol.

[8] Colin P, Ouzzane A, Pignot G, Ravier E, Crouzet S, Ariane MM (2012). Comparison of oncological outcomes after segmental ureterectomy or radical nephroureterectomy in urothelial carcinomas of the upper urinary tract: results from a large French multicentre study. BJU Int.

[9] Takayanagi A, Masumori N, Takahashi A, Takagi Y, Tsukamoto T (2012). Upper urinary tract recurrence after radical cystectomy for bladder cancer: incidence and risk factors. Int J Urol.

[10] Pak RW, Moskowitz EJ, Bagley DH (2009). What is the cost of maintaining a kidney in upper-tract transitional-cell carcinoma? An objective analysis of cost and survival. J Endourol.

[11] Giannarini G, Schumacher MC, Thalmann GN, Bitton A, Fleischmann A, Studer UE (2007). Elective management of transitional cell carcinoma of the distal ureter: can kidney-sparing surgery be advised?. BJU Int.

[12] Montie JE, Clark PE, Eisenberger MA, El-Galley R, Greenberg RE, Herr HW (2009). Bladder cancer. J Natl Compr Canc Netw.

[13] Wein A (2012). Campbell-walsh urology.

[14] Eble JN, Sauter G, Epstein Jl, Sesterhenn I (2004). WHO classification of classification of tumours of the urinary system and male genital organs.

[15] Sobin LH, Gospodarowicz MK, Wittekind C (2009). TNM classification of malignant tumors. UICC International Union against Cancer.

[16] Zhang Z, Zhang C, Wu C, Yang B, Wang H, Hou J (2015). Progressive ureteral dilations and retrograde placement of single-j stent guided by flexible cystoscope for management of ureteroenteral anastomotic stricture in patients after radical cystectomy and bricker urinary diversion. J Endourol.

[17] Ho KL, Chow GK (2005). Ureteroscopic resection of upper-tract transitional-cell carcinoma. J Endourol.

[18] Pohar KS, Sheinfeld J (2001). When is partial ureterectomy acceptable for transitional-cell carcinoma of the ureter?. J Endourol.

[19] Li WM, Wu WJ, Li CC, Ke HL, Wei YC, Yeh HC (2013). The effect of tumor location on prognosis in patients with primary ureteral urothelial carcinoma. Urol Oncol.

[20] Romero FR, Muntener M, Permpongkosol S, Kavoussi LR, Jarrett TW (2006). Laparoscopic-assisted nephroureterectomy after radical cystectomy for transitional cell carcinoma. Int Braz J Urol.

[21] Sanderson KM, Cai J, Miranda G, Skinner DG, Stein JP (2007). Upper tract urothelial recurrence following radical cystectomy for transitional cell carcinoma of the bladder: an analysis of 1069 patients with 10-year followup. J Urol.

[22] Kenworthy P, Tanguay S, Dinney CP (1996). The risk of upper tract recurrence following cystectomy in patients with transitional cell carcinoma involving the distal ureter. J Urol.

[23] Picozzi S, Ricci C, Gaeta M, Ratti D, Macchi A, Casellato S (2012). Upper urinary tract recurrence following radical cystectomy for bladder cancer: a meta-analysis on 13,185 patients. J Urol.

